# Influence of Pre-Dispersion Media on the Batch Reactor Dissolution Behavior of Al_2_O_3_ Coated TiO_2_ (NM-104) and Two ZnO (NM-110 and NM-111) Nanomaterials in Biologically Relevant Test Media

**DOI:** 10.3390/nano12030566

**Published:** 2022-02-07

**Authors:** Else Holmfred, Jens J. Sloth, Katrin Loeschner, Keld Alstrup Jensen

**Affiliations:** 1 National Research Center for the Working Environment, 2100 Copenhagen, Denmark; 2 Research Group for Analytical Food Chemistry, Division of Food Technology, National Food Institute, Technical University of Denmark, 2800 Kongens Lyngby, Denmark; jjsl@food.dtu.dk (J.J.S.); kals@food.dtu.dk (K.L.)

**Keywords:** pre-dispersion, nanomaterials, ICP-MS, dissolution, biosimulant fluids

## Abstract

Dissolution plays an important role on pulmonary toxicity of nanomaterials (NMs). The influence of contextual parameters on the results from dissolution testing needs to be identified to improve the generation of relevant and comparable data. This study investigated how pre-dispersions made in water, low-calcium Gamble’s solution, phagolysosomal simulant fluid (PSF), and 0.05% bovine serum albumin (BSA) affected the dissolution of the Al_2_O_3_ coating on poorly soluble TiO_2_ also coated with glycerine (NM-104) and rapidly dissolving uncoated (NM-110) and triethoxycaprylsilane-coated ZnO (NM-111) NMs. Dissolution tests were undertaken and controlled in a stirred batch reactor using low-calcium Gamble’s solution and phagolysosomal simulant fluid a surrogate for the lung-lining and macrophage phagolysosomal fluid, respectively. Pre-dispersion in 0.05% BSA-water showed a significant delay or decrease in the dissolution of Al_2_O_3_ after testing in both low-calcium Gamble’s solution and PSF. Furthermore, use of the 0.05% BSA pre-dispersion medium influenced the dissolution of ZnO (NM-110) in PSF and ZnO (NM-111) in low-calcium Gamble’s solution and PSF. We hypothesize that BSA forms a protective coating on the particles, which delays or lowers the short-term dissolution of the materials used in this study. Consequently, the type of pre-dispersion medium can affect the results in short-term dissolution testing.

## 1. Introduction

Data on the solubility and dissolution rates of manufactured nanomaterials (NMs) and their potential phase transformation in biological systems have received increasing interest during the last few years. The increased interest is not least due to recent demands for this type of information for regulatory read-across and grouping in chemicals registration and risk assessment and for assessing the potential uptake and accumulation in the human body [1,2]. The solubility and dissolution rates of NMs are important parameters controlling their potential toxicological reaction paths [3,4]. Consequently, dissolution testing in biosimulant fluids can be an important measure for understanding the dissolution behavior to estimate potential harmful effects.

Solubility and dissolution testing can be made using several different procedures and test media. Conceptually, however, there are three approaches (static, dynamic, and sequential dissolution) that each have their strengths and weaknesses. To understand and apply the results well and allow mutual acceptance of data, it is necessary to harmonize key conceptual parameters and procedures in the specific test methods as much as possible to allow the generation of comparable and reliable data. Therefore, it is necessary to identify the critical steps and parameters in the protocols applied and understand to what extent they should be defined or controlled.

In this work, we investigate the potential need to harmonize the pre-dispersion step in a protocol for short-term (≤24 h) stirred batch reactor dissolution testing. NMs are often supplied as powders, and creating liquid suspension of NMs is a step required for several analyses and tests, including size analysis (e.g., Verleysen et al. [5]), dissolution testing in batch systems (Holmfred et al., [6]), and many (eco-)toxicological studies (intratracheal installation, pharyngeal aspiration, and injection) [7,8,9]. In most cases, such NM dispersions are prepared in stock suspensions [7,10]. Preparation of a stock suspension has the advantage of creating a dispersion with potentially well-controlled and measurable particle sizes and zeta potential (depending on the dispersion medium and NM) allowing adequate dosing precision. A potential drawback of working from an NM stock suspension is that it may cause artifacts in the test due to changes in material characteristics and properties or reactions or interactions in or with the liquid media before analysis or testing is started [7]. Hadrup et al. investigated the influence of a pre-dispersion medium on pulmonary inflammation induced by NMs in rats [11]. Carbon black, TiO_2_, and carbon nanotubes were used as model NMs, and the materials were dispersed in water, 2% serum, 0.05% serum albumin in water, 10% bronchoalveolar lavage fluid (in 0.9% NaCl or water), or 0.1% Tween-80 in water [11]. The acute inflammation was shown to be media-dependent for carbon black as DNA damage was only observed after dispersion with water, 2% serum, and 10% bronchoalveolar lavage fluid in 0.9% NaCl, whereas for TiO_2_, no genotoxicity effects were dependent on the dispersion medium used. Conclusively, Hadrup et al. suggested to consider the type of dispersion medium when using intratracheal instillation exposure [11]. In addition, Sauer et al. [12] investigated the NM agglomeration and biological effects of different dispersion media. In their study, 16 different NMs, including ZnO, Ag, TiO_2_, CeO_2_, SiO_2_, and carbon nanotubes, were investigated. The NM dispersions were prepared in porcine lung surfactant and bovine serum albumin and shown to influence the effective dosage after 12 and 24 h. Overall, using bovine serum albumin as a dispersant created stable and narrow size-distribution dispersions; however, porcine lung surfactant resembles more closely the proteins in the lung-lining fluid [12].

The quality of the pre-dispersion is critical for achieving reproducible size-analysis (within sub- to micron meter size reflecting the approximate minimum aggregates size) and dosing as well as for enhancement of surface-area-related processes, size-related uptake, and translocation. Relevant for dissolution testing of particles, size and accessible surface area are considered critical physicochemical parameters affecting the dissolution kinetics of NMs [4,13]. To create a reproducible liquid NM dispersion, considerations of several parameters such as choice of the dispersion medium, need for adjustment of pH and ionic strength, and de-agglomerating energy (ultrasonication, stirring, and shaking) must be made as these parameters influence the final degree of the dispersion [7,8,14,15]. The role of accessible surface area in the dissolution of particle agglomerates has been described by David et al. [16].

A study of the dissolution rates in different test media used for simulating a pulmonary compartment using a continuous flow-through membrane method has clearly shown important differences in the rates and dissolved amounts depending on the media [15]. Consequently, the properties of the dispersion medium might play a role in the possible dissolution of the NM prior to the actual dissolution test, e.g., during sampling. Finally, it is also important to investigate the extent to which specific protocol steps in dissolution testing, for example, the use of dispersion agents such as proteins, affect the NM solubility and dissolution rates. Protein adsorption is verified to occur on many NMs when present in dispersion and in vitro test media and are thought to be important determinants in NM interaction with biological compartments [17,18]. Understanding the potential effects of these factors is relevant as new test methods aim to become more biologically relevant and biomolecules such as proteins and enzymes are naturally present in biological systems.

In this study, we studied the role of different pre-dispersion media on dispersibility and dissolution rates using three well-characterized and industrially representative nanomaterials (organically uncoated and coated ZnO (NM-110 and NM-111) and organic-inorganic coated TiO_2_ (NM-104)). These materials have different surface properties and aggregate structures and known differences in solubilities and dissolution rates [17,19,20]. We hypothesize that the type of dispersion medium and presence of protein as dispersant will influence the short-term dissolution kinetics of NMs. This knowledge may be important for further protocol development and interpretation of dissolution tests and toxicological studies if differences occur. Consequently, this study aims at investigating the dissolution behavior of ZnO (NM-110 and NM-111) and Al_2_O_3_-coated TiO_2_ (NM-104) in two biosimulants as test media using water, test media, and 0.05% *w*/*v* bovine serum albumin (BSA) water as pre-dispersion media. The test media were low-calcium Gamble’s solution, a surrogate for the lung-lining fluid [21], and Phagolysosomal Simulant Fluid (PSF), a surrogate for the lung alveolar macrophage phagolysosomal fluid [22].

## 2. Materials and Methods

### 2.1. Test Materials

TiO_2_ (NM-104) and ZnO (NM-110 and NM-111) originated from the OECD Working Party on Manufactured Nanomaterials Sponsorship Programme and were all obtained from the Fraunhofer Institute for Molecular Biology and Applied Ecology (Schmallenberg, Germany). The two ZnO materials represent a group of relatively rapid dissolving materials of which ZnO (NM-111) is organically coated with hydrophobic triethoxycaprylsilane, and NM-104 represents a poorly soluble NM coated with Al_2_O_3_ and glycerine [20]. The key physicochemical characteristics of the test materials are summarized in Table 1. Samples were stored under argon until subsampling for testing.

### 2.2. Pre-Dispersion of Nanomaterials

The nanomaterials were pre-dispersed at the same acoustic power of 7.35 ± 0.05 Watt following the so-called NANOGENOTOX batch dispersion protocol validated as part of the FP7 NANoREG project [10]. The pre-dispersion media were either 0.05% BSA (standard protocol medium), ultrapure water (resistivity 18 MΩ·cm) (Nanopure^®^ system, Thermo Fisher Scientific, Waltham, MA, USA), or test media (PSF and low-calcium Gamble’s solution). The 0.05% BSA was prepared using ultrapure water. PSF and low-calcium Gamble’s solution was prepared as described below.

Dispersions were prepared by weighing 37.5 mg of NM into a 15 mL glass vial. Powders were pre-wetted with 0.5% *w/v* 96% ethanol and dispersed with 14.57 mL of solution (0.05% BSA, water, or test media) to a final concentration of 2.56 mg/mL. A 400 W Branson Sonifier S-450D (Branson Ultrasonics Corp., Danbury, CT, USA) equipped with a 13 mm disruptor horn was used to sonicate the particle dispersion for 16 min with a 10% amplitude (approx. 42 W) under constant cooling in an ice-water bath, adding the required acoustic delivered power. Ethanol pre-wetting and the same sonication conditions were applied for all pre-dispersion media to allow dispersion of both hydrophilic (NM-104 and NM-110) and hydrophobic (NM-111) test materials. It should be noted that all results concerning pre-dispersion in 0.05% BSA water as dispersion medium were obtained from Holmfred et al. [6].

### 2.3. Evaluation of the Dispersion Quality and Stability

The quality and stability of the particle dispersions were evaluated using a Malvern Zetasizer Nano ZS (Malvern Panalytics Ltd., Malvern, UK) measuring the hydrodynamic size distribution (Z_ave_), zeta potential (Z_pot_), and polydispersity index (PDI). The instrument was equipped with a 633 nm laser measuring at 173° (non-invasive backscattering). Immediately after preparation of the dispersion, 700 µL was transferred to a disposable folded capillary cell (DTS1070, Malvern). Prior to analysis, the instrument was equilibrated for 5 min. All samples were analyzed using automatic optimization mode of measurement of configuration, at 25 °C using the viscosity of water (0.8872 cP), and optical parameters of water. Z_ave_ and PDI were reported as an average of ten repeated measurements. The Z_pot_ was measured using the Smoluchowski model calculated in automatic mode based on ten repeated measurements. The obtained average Z_ave_ values were compared with scanning electron microscopy aggregate sizes and Z_ave_ benchmark sizes generated by DLS to qualify dispersions made using the NANOGENOTOX dispersion protocol [10].

### 2.4. Albumin Adsorption

The amount of adsorbed BSA to the TiO_2_ (NM-104) and ZnO (NM-110 and NM-111) was quantified from ELISA reader analysis of total protein concentration in the 0.05% BSA batch dispersion medium after sonication with each of the three test materials using the Pierce^TM^ BCA Protein Assay Kit (Thermo Scientific, Rockford, IL, USA, catalog no. 23227). A total of 1 mL dispersion medium was sampled and added to an Eppendorf tube and centrifuged for 30 min at 20,000 RCF to settle the particles and avoid interference in the ELISA reader. The particle-free supernatant was then sampled, allowed to react with the reagent from the kit in 96-well plates, and analyzed against a BSA calibration curve in the ELISA fluorimeter. All ELISA determinations were based on double tests, and concentrations were derived from BSA calibration curves. Concentrations measured in samples of particle-free dispersion medium were used for reference. The amount of adsorbed BSA was calculated by ascribing the difference between the BSA concentrations in the dispersions with and without sample to adsorption by the particles. Data are given as the BSA-load on the particles (µg BSA/mg particle) by dividing the measured BSA concentration difference by the batch particle concentration of 2.56 mg/mL and the percent adsorbed of added BSA.

### 2.5. Physiological Simulant Test Fluids

Phagolysosomal simulant fluid (PSF) and low-calcium Gamble’s solution were prepared by dissolving the components listed in Table 2 in 2 L ultrapure water (<18 MΩ·cm) (Thermo Fisher Scientific, Waltham, MA, USA), respectively. The solutions were left overnight and filtered the following day through a polyvinylidene fluoride membrane 0.45 µm filter (Merck Millipore Ltd., Tullagreen, Ireland). PSF and low-calcium Gamble’ solution have a shelf-life of approx. 1–1.5 months when stored at 5 °C protected from light. The pH of the PSF and low-calcium Gamble’s solution were approximately 4.5 and 7.4 after preparation, respectively. All chemicals were purchased from Merck (Darmstadt, Germany).

### 2.6. Dissolution Testing in Stirred Batch Reactors

Dissolution testing was completed in an atmosphere–temperature–pH-controlled (ATempH) stirred batch reactor (SBR) system described and validated in Holmfred et al. [6]. In brief, the system consists of four reactors, each coupled to an OMNIS titration system (Metrohm, Herisau, Switzerland); one batch reactor was used as a reference containing pure test medium (low-calcium Gamble’s solution or PSF), and three batch reactors were used for a test of NMs (n = 3). To all reactors, 96 mL of test medium was added before testing. Throughout testing, pH, temperature, and gas flows are kept constant. To mimic physiological lung conditions, the temperature is set to 37 °C, and O_2_ and CO_2_ flow are set to 144 and 5.62 mL/min, respectively. Before testing, low-calcium Gamble’s solution and PSF were adjusted with 0.1 M NaOH or HCl to exactly pH 7.4 and 4.5, respectively, and kept constant throughout testing. The particle suspension was transported gently from the probe sonicator, and 4 mL of suspension was added to each of the test reactors at the start of the test resulting in a nominal NM concentration of 102.4 mg/L. To the reference reactor, 4 mL of the respective pre-dispersion medium was added. At t_0_, t_1_, t_2_, t_4_, and t_24_ hours, 4 mL sample was collected using a 5 mL plastic syringe with a steel needle and transferred to 3 kDa Amicon Ultra-4 centrifugal filters (Merck, Darmstadt, Germany) and immediately centrifuged for 30 min at 4000× *g*, 4400 rpm using an RF+ Sorvall centrifuge (Thermo Fisher Scientific, Waltham, MA, USA). The time from finalising the pre-dispersion and to the t_0_ h samples were in the centrifuge was approximately 2 min. After centrifugation, the filters were removed, and 0.5 mL of 2% nitric acid was added for stabilization of the dissolved ionic fraction.

### 2.7. Dissolved Ionic Fraction

The dissolved ionic fraction was diluted 4–1000 times with 2% HNO_3_ and measured using inductively coupled plasma-mass spectrometry (ICP-MS), as specified in Table 3. The total concentrations of dissolved Zn (NM-110 and NM-111) as well as Ti and Al (NM-104) were measured at the sampling time points t_0_, t_1_, t_2_, t_4_, and t_24_ using a Thermo iCAP SQ ICP-MS (Thermo Fisher Scientific, Waltham, MA, USA) equipped with an ASX-560 autosampler (Teledyne Cetac Technologies, Omaha, NE, USA). The ICP-MS was further equipped with a Thermo Fisher Scientific quarts cyclonic spray chamber and a PFA-ST MicroFlow nebulizer. During analyses, the plasma power was 1550 W, the plasma gas flow was 14.00 L/min, and the dwell time was set to 100 ms. ICP-MS parameters can be found in Table 3.

The 3 kDa filtrates were diluted to an acidic concentration of 2% nitric acid and were quantified against an external calibration curve prepared in 2% nitric acid. Considering the dissolution of TiO_2_ (NM-104) in PSF, the total ionic fraction was quantified against an external calibration curve prepared in 10 times diluted PSF having the same dilution factor as the analyzed samples. ICP-MS certified stock standard solutions 1000 mg/L (SCP SCIENCE, Quebec, Canada) with trace elements ≤ 1 µg/L were used to prepare all external calibration curves. The internal standards were likewise prepared in 2% nitric acid from ICP-MS standards (100 mg/L) and diluted to a final concentration of 20 µg/L and added on-line to the sample flow using a T-piece. Blanks and spiked samples were included in all analyzes for quality control of the ICP-MS. The concentrations determined in the reference reactor were subtracted from the measured concentrations of the samples. To avoid carry-over, a rinsing procedure with 2% nitric acid was performed between all sample injections. The limit of detection (*LOD*) of the measured element in the filtrated sample was calculated using Equation (1)

(1) LOD=3·SD·DF,

where *SD* is the standard deviation of the concentration of a minimum of five blank samples, and *DF* is the dilution factor.

### 2.8. Determination of Initial Dissolution Rates

At each sampling time point (t_0_, t_1_, t_2_, t_4_, and t_24_ hours), the measured dissolved ionic fractions were multiplied with the total dilution factor, corrected for moisture content, coating, and impurities (Table 1), and stoichiometrically adjusted to obtain the dissolved concentrations and remaining fractions of ZnO and TiO_2_, respectively. The total dilution factor includes the dilution volume from acid/base titration during 24 h of testing and dilution with 0.5 mL 2% nitric acid to stabilize the sample filtrates.

Previous studies have described that the dissolution of most NMs follow first-order kinetics [13,28,29]. However, in our data, it was impossible to identify any clear 0., 1., and 2. order kinetics using the integral method for determining the dissolution rates [30,31]. Dissolution rates were therefore estimated using the numerical differential method [30,31]. In short, a non-linear regression function was established for the obtained elemental concentrations as a function of time following Equation (2):

(2) $$C\left(t^{*}\right)=\theta_{1}-\theta_{2}\cdot \exp\left(-\theta_{3}\cdot t^{*}\right),$$

where C(t*) is the concentration as the function of time, t^*^ is the adjusted time, and $\theta_{1},\;\theta_{2}$, and $\theta_{3}$ are constant fitting parameters of the non-linear equation obtained by the plot of concentration and time. Equidistant real time-points were calculated by taking the 16 min of sonication, sampling, and filtration into account, Equation (3)

(3) $$t^{*}=t_{sampling}+25\;\min,$$

The 25 min time adjustment was made as an approximation of the total effective delay related to sonication, dosing, sampling, and effective filtration time. The *t_sampling_* was slightly different for each sample (few seconds) as the samples were collected manually. The centrifuge is kept running for 30 min to ensure that all possible liquid is removed from the filter membrane. However, >75% of the liquid is filtered through within <10 min, according to technical information from Amicon. Using the adjusted time-points, initial, intermediate, and last points were determined according to Fogler (1999) [30]

(4) $$Initial:\;{\left(\frac{dC_{A}}{dt}\right)}_{t_{0}}=\frac{-3C\left(t=0\right)+4C\left(t=1\right)-C\left(t=2\right)}{2∆t}$$

where *C_A_* is the concentration

(5) $$Intermediate:\;{\left(\frac{dC_{A}}{dt}\right)}_{t}=\left(\frac{1}{2∆t}\right)\left[C\left(t=t+1\right)-C\left(t=t-1\right)\right]$$

(6) $$Last:\;{\left(\frac{dC_{A}}{dt}\right)}_{t_{end}}=\left(\frac{1}{2∆t}\right)\left[C\left(t=t_{end}-2\right)-4C\left(t=t_{end}-1\right)-3C\left(t=t_{end}\right)\right]$$

The plot of the numerical points as a function of t* were used to determine the initial dissolution rate projected at *t* = 0, ${\left(\frac{dC_{A}}{dt}\right)}_{t=0}$. The surface area dissolution rate was determined by Equation (7):

(7) $${\left(\frac{dC\left(SSA\right)}{dt}\right)}_{t=0}={\left(\frac{dC_{A}}{dt}\right)}_{t=0}\cdot SSA$$

where ${\left(\frac{dC_{A}}{dt}\right)}_{t=0}$ is the initial dissolution rate and *SSA* is the specific surface area measured by the Brunauer–Emmett–Teller method.

### 2.9. Statistics

Two-way repeated measures ANOVA (analysis of variance) was used to test for independence between dissolution rates and type of pre-dispersion medium. The independence was tested across all measured concentrations (at time points t_0_, t_1_, t_2_, t_4_, and t_24_ hours). *p*-value ≤ 0.05 was considered significant. Measurements at different time points within a replication were treated as repeated measurements with a first-order autoregressive correlation structure and a model-based covariance matrix. The analyses were conducted by use of the mixed procedure in SAS version 9.4 statistical software (SAS Institute Inc., Cary, NC, USA).

## 3. Results and Discussion

### 3.1. Albumin Adsorption

The results from analysis of BSA adsorption onto TiO_2_ (NM-104) and ZnO (NM-110 and NM-111) are listed in Table 4. Two rounds of tests are shown, for all of which a second set of data on ZnO (NM-110 and NM-111) was obtained from Da Silva et al. (2019a) [17]. The results show that a high amount of BSA adsorbs onto all three materials, yielding a theoretical mass-based coverage ranging between 40% and 78% of the BSA present in the 0.05% BSA pre-dispersion medium. The highest amounts of adsorption were observed on ZnO (NM-110) and TiO_2_ (NM-104). Slightly lower levels of adsorption were found for the triethoxycaprylsilane coated ZnO (NM-111), probably as the organic coating reduces the binding to BSA. The difference in BSA-adsorption observed for ZnO (NM-110 and NM-111) is in agreement with the listed results obtained for the NANOGENOTOX dispersion protocol in Da Silva et al. (2019a) [17]. Previous work has also documented BSA adsorption onto TiO_2_ (NM-104) and other TiO_2_ nanomaterials using a tailored BSA-enhanced dispersion protocol [19].

### 3.2. Dispersion Quality

Table 5 lists the results from DLS and Z_pot_ measurements on the pre-dispersions of TiO_2_ (NM-104) and ZnO (NM-110 and NM-111) prepared in water, 0.05% BSA, PSF, or low-calcium Gamble’s solution made with the three test materials in water, 0.05% BSA, PSF, and low-calcium Gamble’s solution before dissolution testing in either PSF or low-calcium Gamble’s solution. The individual DLS size distribution spectra are given in the supplemental material (Figures S1–S3). Overall, the results show that pre-dispersion in water and 0.05% BSA provides the smallest Z-average (Z_ave_), while dispersion directly in test media generally results in larger Z_ave_ values. Only TiO_2_ (NM-104) disperses better in water than in 0.05% BSA, which is known to be an effect of increased agglomeration in the presence of BSA [19]. In general, water and test media formed unstable dispersions (30 mV > Z_pot_ > −30 mV) [8], and the 0.05% BSA dispersions were considered stabilized by the protein [7]. However, the highly positive Z_pot_ of ZnO (NM-110) after pre-dispersion in water (24.6 ± 0.6 and 30.7 mV ± 0.6) indicates that a stable suspension is formed. Water could be considered a dispersion medium for this material in a case-to-case based dispersion medium decision.

The level of agglomeration in the different pre-dispersion media was evident when the measured Z_ave_ values were compared to particle average agglomeration sizes (D_aggr_) determined by scanning electron microscopy and benchmark DLS sizes (Z_ave,benchmark_) for the NANOGENOTOX dispersion protocol (Jensen et al. [10]; Mejia et al. [32] and available from the eNanoMapper database [33]) (Table 5). In this comparison, the agglomeration in the test media resulted in 16.0 (ZnO (NM-110) in low-calcium Gamble’s solution) to 58.9 (TiO_2_ (NM-104) in PSF) times larger Z_ave_ sizes than observed in the benchmark DLS data. Good comparability of the dispersion quality parameters listed in Table 5 was observed between the pre-dispersions made in this study using the 0.05%BSA (the standard medium of the NANOGENOTOX disperson protocol) and the benchmark values for the NANOGENOTOX dispersion protocol. Only, the 0.05% BSA dispersion of TiO_2_ (NM-104) used for dissolution testing with low-calcium Gamble’s solution was coarser (ratio = 3.1) compared to all other 0.05% BSA dispersions (ratio 1.1 to 1.6).

### 3.3. Effect of Pre-Dispersion Medium and Quality on Dissolution Behavior

**Figure 1:**
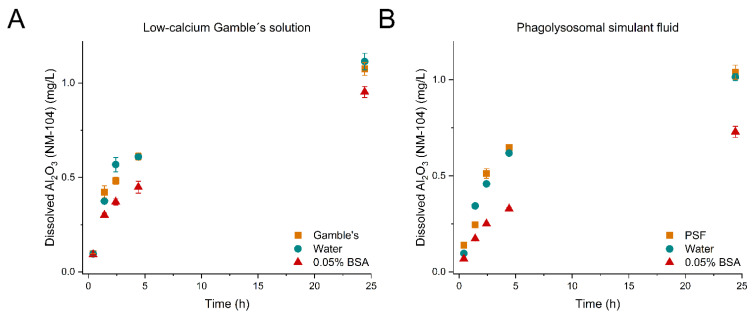
Dissolution profiles of the Al_2_O_3_ coating of TiO_2_ (NM-104) using the ATempH SBR system. (**A**) TiO_2_ (NM-104) dispersed in low-calcium Gamble’s solution (■), water (●), and 0.05% BSA (▲). **(B**) TiO_2_ (NM-104) dispersed in phagolysosomal simulant fluid (PSF) (■), water (●), and 0.05% BSA (▲). The dissolution tests were conducted for 24 h.

**Figure 2:**
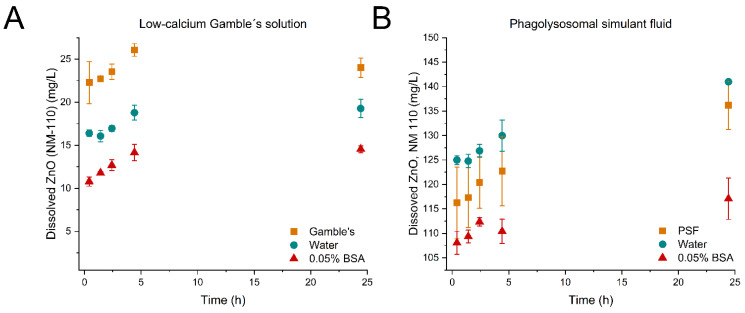
Dissolution profiles of ZnO (NM-110). (**A**) ZnO (NM-110) dispersed in low-calcium Gamble’s solution (■), water (●), and 0.05% BSA (▲). (**B**) ZnO (NM-110) dispersed in phagolysosomal simulant fluid (PSF) (■), water (●), and 0.05% BSA (▲). The dissolution tests were conducted for 24 h. Note the differences in the *y*-axis scale.

**Figure 3:**
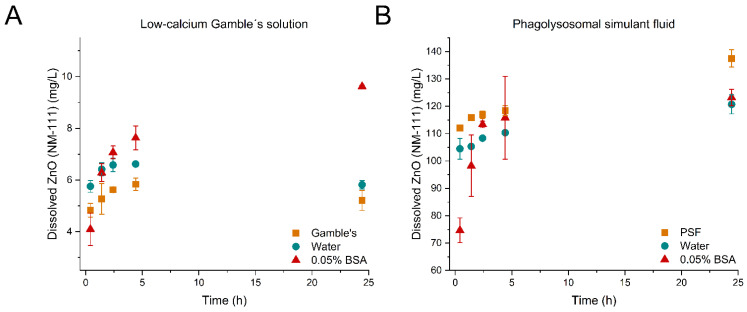
Dissolution profiles of ZnO (NM-111). (**A**) ZnO (NM-111) dispersed in low-calcium Gamble’s solution (■), water (●), and 0.05% BSA (▲). All dispersions were tested in low-calcium Gamble’s solution for 24 h. (**B**) ZnO (NM-111) dispersed in phagolysosomal simulant fluid (PSF) (■), water (●), and 0.05% BSA (▲). All dispersions were tested in PSF for 24 h. Note the differences in the *y*-axis scale.

Figure 1, Figure 2 and Figure 3 show the evolution of dissolved material over time from the dissolution tests with TiO_2_ (NM-104), ZnO (NM-110), and ZnO (NM-111) in low-calcium Gamble’s solution (A) and PSF (B) for the three pre-dispersion media, respectively. *p*-values of the statistical analyses are presented in Table 6.

For TiO_2_ (NM-104), the Al_2_O_3_ coating dissolved gradually over the 24 h test period, while TiO_2_ was not dissolved to concentrations above LOD in any of the tests performed in low-calcium Gamble’s solution and PSF (Figure 1A,B, Table 7). The dissolved Al concentrations reached between 0.036 ± 0.0040 and 0.56 ± 0.051 mg/L in the different tests (0–24 h), corresponding to 0.069 ± 0.0040 – 1.1 ± 0.45 mg/L Al_2_O_3_, and were found to be in the same overall range for testing in both low-calcium Gamble’s solution and PSF (Figure 1 and Table 7). TiO_2_ is known to be practically insoluble in low-calcium Gamble’s solution from a previous static batch dissolution 24 h screening study of the OECD WPMN industrially representative NM of TiO_2_ (NM-100, NM-101, NM-102, NM-103, NM-104, and NM-105) pre-dispersed following the NANOGENOTOX protocol [26]. In the previous study, the Al_2_O_3_ coating on TiO_2_ (NM-103 and NM-104) was observed to dissolve in low-calcium Gamble’s solution and for TiO_2_ (NM-104) reaching 413 µg/L of Al after 24 h (corresponding to 0.780 mg/L Al_2_O_3_ dissolved), comparable to the findings in this study.

We observed that the hydrodynamic size of TiO_2_ (NM-104) was highly affected by the pre-dispersion medium, where water and 0.05% BSA formed dispersions with the lowest Z_ave_ while the particles agglomerated heavily when dispersed in the test media. However, the samples pre-dispersed in water and test media (low-calcium Gamble’s solution or PSF) showed comparable dissolution profiles (*p*-value: 0.5233 or *p*-value: 0.6167, respectively) with a significantly 1.2–1.3 fold higher amount of total dissolved Al_2_O_3_ after 24 h than after pre-dispersion in 0.05% BSA (Figure 1A,B). The dissolution profiles after dispersion with 0.05% BSA were significantly different from the water and test media dispersions (*p*-values in Table 6); however, the initial dissolution rates were not affected by the choice of pre-dispersion medium (Table 7). Therefore, a direct agglomerate size effect on dissolution was not observed in the dissolution of Al_2_O_3_ (NM-104). A total of 131.8 ± 22.0 µg BSA/mg was found to adsorb to TiO_2_ (NM-104) (Table 4); the protein adsorption appeared to affect the dissolution of Al_2_O_3_ at neutral (low-calcium Gamble’s solution) and acidic pH (PSF).

Testing of ZnO (NM-110) dissolution in low-calcium Gamble’s did not show a significant influence of the pre-dispersion medium (*p*-values in Table 6) on the dissolution kinetics as the dissolution profiles over 24 h were only found to be shifted parallel (Figure 2A). However, the pre-dispersion medium seems to affect the starting concentrations, i.e., the amount of material dissolved during sonication, sampling, and filtration and/or the NM dose, but the initial dissolution rates were comparable.

The pre-dispersion medium affected the dissolution of ZnO (NM-110) in PSF (Figure 2B). No significant difference was observed between water and PSF as a pre-dispersion medium (*p*-value: 0.5987), and the initial dissolution rates were comparable (Table 7). However, the dissolution of ZnO in PSF was affected by using 0.05% BSA compared to water (*p*-value: 0.0141) and PSF (*p*-value: 0.0267) as a pre-dispersion medium. The ZnO (NM-110) concentrations after pre-dispersion in PSF showed large standard deviations throughout the testing. The measured concentrations exceeded the nominal dose of 102.4 mg/mL for all three pre-dispersion media. Inhomogeneous particle dispersions and/or variability in the dosing could explain this observation. Homogeneous suspensions and pre-dispersion stability are critical for accurate dosing [7], as the factors can directly affect the dissolution behavior and the batch-to-batch variation of dissolved material. Generally, the solubility of ZnO (NM-110) increased approximately 10-fold in PSF compared to low-calcium Gamble’s solution. Despite the minor differences in the compositions of the complex lung simulants (Table 2), the solubility of Zn^2+^ ions increases with lower pH [2]. Rapid dissolution of ZnO has also been demonstrated previously by Keller et al. [15]. The extremely rapid dissolution in PSF occurs within seconds from addition to the ATempH SBR system. The protein adsorption of BSA (138.3 ± 18.4 µg BSA/mg ZnO) to ZnO (NM-110) may slow this extreme dissolution rate, but with the current setup of the ATempH SBR system, it was impossible to determine the initial rates of ZnO (NM-110) in PSF.

The coated ZnO (NM-111) demonstrated an expected lower solubility in low-calcium Gamble’s solution than in PSF (Figure 3A,B) due to the pH difference [2]. The variation across the three batch reactors resulted in large standard deviations of the 0.05% BSA dispersion of ZnO (NM-111) (Figure 3B). Reasonably, the variation could be assigned to dosing and sampling differences. Compared to results from testing ZnO (NM-110), ZnO (NM-111) in low-calcium Gamble’s solution unexpectedly showed lower solubility after 24 h despite similar initial dissolution rates when pre-dispersed in the 0.05% BSA Table 7, Figure 3A). The different dissolution behavior of ZnO (NM-110) and ZnO (NM-111) in the presence of 0.05% BSA might be related to the additional presence of an organic coating on ZnO (NM-111). The dissolution using 0.05% BSA was significantly different from the dispersions in low-calcium Gamble’s solution (*p*-value: <0.0001) and water (*p*-value: 0.0711), while there was no difference between low-calcium Gamble’s solution and the water dispersion (*p*-value: 0.2858).

Despite the organic coating on ZnO (NM-111), the dissolution in PSF occurred immediately (pre-dispersion in water or PSF) or within the first hour (pre-dispersion with 0.05% BSA) (Figure 3B); therefore, it was impossible to determine the initial dissolution rates. Statistically, the dissolution was affected by all three pre-dispersion media (*p*-values in Table 6); there was a difference between using water and PSF as a dispersion medium. However, this might be a size effect, as the PSF dispersion formed extremely large agglomerates. A lower initial dissolved concentration (t_0_ + 25 min) of the 0.05% BSA dispersion was potentially influenced by particle–protein interaction as 88.3 ± 27.1 µg BSA/mg ZnO (NM-111) was found adsorbing to the surface (Table 4). In all cases, the dissolved amount of ZnO (NM-111) reached more than 100% dissolved material (i.e., dissolved concentrations > nominal concentration of 102.4 mg/L), likely resulting from inhomogeneous particle dispersion and the important, though critical, addition of particles to the test batch reactors.

The observed initial suppression of Al-oxide and Zn dissolution using 0.05% BSA may be due to different mechanisms. We identified that a large amount of the BSA in the 0.05% BSA adsorb to the test materials (Table 4). However, dissolved Al^3+^ and Zn^2+^ may also adsorb or bind to BSA [34], suppressing the concentration of free ions [35]. We describe two scenarios for the suppressed release of Al^3+^ and Zn^2+^ after dispersion with 0.05% BSA.

**Scenario 1:** Studies have reported that albumin can form mono and polynuclear complexes with metal ions as Zn^2+^, Cu^2+^, Pt^2+^, Cu^2+^, Ni^2+^, and Co^2+^ [34]. Complexation of Al^3+^ or Zn^2+^ to BSA could potentially happen on the surface of the particles as well as with ions released from the surface, forming complexes in the solution. In the case of TiO_2_ (NM-104), based on the assumption that all 6 wt.% Al_2_O_3_ coating is completely released from the material, aluminum will be in great excess (10^5^ fold by molarity) compared to the BSA. One could expect that complexation could lower the concentration of free and measurable ions. The size of BSA has been reported as 66 kDa [36], whereas the used filters are a 3 kDa membrane. In the case of ion–BSA complexation, the complexes will be trapped and cleared by the filtration, thereby decreasing the determined dissolution output. However, the initial dissolution concentrations were comparable in all cases of dissolving Al_2_O_3_ (Table 7), indicating that potential Al–protein complexation has negligible importance in these tests. As ZnO (NM-110 and NM-111) fully dissolves in PSF, the hypothesis of Zn-BSA complexation was also not found plausible for Zn^2+^ ions.

**Scenario 2:** An alternative hypothesis to the observed suppressed release of ions after dispersion with 0.05% BSA is capping or “surface coating” of the NM. Dispersion in water, low-calcium Gamble’s solution, and PSF all demonstrate a higher release of aluminum (despite the differences in pH) compared to 0.05% BSA dispersions, which supports the assumption of the BSA binding to the surface of the TiO_2_ (NM-104) particles. Vergaro et al. investigated the interaction between nanosized TiO_2_ and human serum albumin and found that serum albumin interacts with TiO_2_ nanocrystals [37].

A complete understanding of the relative role of BSA as a protective capping or ion scavenger requires further in-depth studies to make clear conclusions. However, at the state of writing, we consider Scenario 2 the most likely explanation for why the Al^3+^ and Zn^2+^ dissolution is suppressed after dispersion with 0.05% BSA (Figure 1, Figure 2 and Figure 3).

### 3.4. Pro et Con Analysis Regarding the Selection of Pre-Dispersion Media for Dissolution Testing

The purpose of creating homogeneous liquid pre-dispersions of test materials as an initial step in batch reactor dissolution testing is to allow more precise dosing [8] and at least reasonable suspension of dispersed particles during dissolution testing. Direct dosing of dry powders to batch reactors cannot be applied due to high differences in, e.g., agglomeration and hydrophobicity of NMs. However, the liquid pre-dispersion also has drawbacks as dissolution starts as soon as the particles are added to the dispersion media. As seen in the case for both TiO_2_ (NM-104) in low-calcium Gamble’s solution and PSF (Figure 1A,B), ZnO (NM-110) in PSF (Figure 2B) and ZnO (NM-111) in low-calcium Gamble’s solution and PSF (Figure 3A,B), the dissolution over 24 h was affected by the type of pre-dispersion medium.

Overall, the most pronounced effect on dissolution behavior was seen by using the 0.05% BSA as a dispersion medium, which resulted in generic good dispersions but delayed the dissolution, as discussed earlier. A total of 0.05% BSA was initially chosen in NANOGENOTOX as a biologically acceptable dispersion medium, providing reasonable dispersability and ability to stabilize particles through different mechanisms (electric, steric, and depletion stabilization) [10]. Pre-dispersion by use of protein dispersants satisfy the needs to perform harmonized in vitro and in vivo toxicological testing as applied in several EU projects [7]. The stock dispersions require a high enough concentration to depict the potential toxic effect and meet the detection limits. As seen in this study, 0.05% BSA suppressed the dissolution signal potentially driven by a complex or capping effect by the protein.

In general, the use of proteins as a stabilization agent of the particle dispersion reflects the conditions in biological systems better. Further, it is an advantage of the observed delayed dissolution that it actually allows better measurement of the initial dissolution rate. While BSA is a relevant biological protein, the observation raises a question about whether a generic dispersion protocol with proteins is the best option to perform dissolution studies or dispersion should be based on a case-by-case decision. The advantage of a generic protocol is comparability between materials, repetitions, and laboratories. The choice of pre-dispersion medium may be steered by relevance for whether a biological or non-biological compartment is tested. However, a generic protocol can reach a limit, as not all NMs may be adequately dispersed using the same liquid dispersion medium. As seen in this study, the choice of dispersion medium potentially can affect the starting concentration and dissolution rates. Therefore, harmonization is required to reach good comparability between experiments and laboratories. Well-justified modifications could be accepted case-by-case. The protocol can also be improved in the future by adding measurements of initial particle concentrations, which would enhance the ability for quality control and increase the comparability of individual experiments.

## 4. Conclusions

This study demonstrated that pre-dispersion is an important parameter to consider in batch reactor dissolution testing of NMs. We showed that the choice of the pre-dispersion medium could significantly affect the dissolution profiles of TiO_2_ (NM-104) and ZnO (NM-111) in low-calcium Gamble’s solution and PFS and ZnO (NM-110) in low-calcium Gamble’s solution over 24 h of testing. The effect of using different pre-dispersion media shows that the choice of pre-dispersion media should be harmonized to allow the generation of comparable results between tests and across laboratories.

## Figures and Tables

**Table 1:** Physicochemical characteristics of the test materials.

Characteristics	NM-104	NM-110	NM-111
Primary particle size ECD * (nm)	25.0 ± 1.7 ^a^	75.4 ± 58.4 ^b^	40.6 ^c^
Average aggregate size ECD * (nm)	58.5 ± 46.3 ^a^	114 ± 97 ^d^	106 ± 69 ^d^
Crystallite size by XRD (nm)	27 ^e^	42 ^f^	24–42 ^f^
Specific surface area (m^2^/g)	56.8 ± 0.5 ^e^	12.4 ± 0.6 ^d^	15.1 ± 0.6 ^d^
Material	TiO_2_—Rutile ^e^	ZnO—Zincite ^f^	ZnO—Zincite ^e^
Inorganic coating (wt.%)	6.08 Al_2_O_3_^g^	None ^f^	None ^e^
Organic coating (wt.%)	3.17 ± 0.07 ^h^ glycerine ^h^	ND ^f^	2.1 ± 0.31 ^g^ triethoxycaprylsilane ^h^
Moisture content (wt.%)	1.50 ± 0.10 ^g^	0.28 ± 0.11 ^g^	ND ^g^

* ECD: Equivalent Circular Diameter, obtained by EDS on sample pellet; ND: not detected. ^a^ De Temmerman et al. (2012), NANOGENOTOX Deliverable 4.2 [23]. ^b^ OECD—Dossier on Zink Oxide (2015) [24]. ^c^ Llewellyn et al. (2021) [25]. ^d^ Da Silva et al. (2019) [17]. ^e^ Rasmussen et al. (2014) [26]. ^f^ Singh et al. (2011) [27]. ^g^ Holmfred et al. (2022) [6]. ^h^ Clausen et al. 2019 [20].

**Table 2:** Composition of phagolysosomal simulant fluid (PSF). Adapted from Ref. [22], and low-calcium Gamble’s solution. Adapted from Ref. [21].

Simulated Lung Fluid	Component	Chemical Formula	Concentration [mg/L]
Phagolysosomal simulant fluid (PSF)	Sodium phosphate dibasic anhydrous	Na_2_HPO_4_	142
	Sodium chloride	NaCl	6650
	Sodium sulphate anhydrous	Na_2_SO_4_	71
	Calcium chloride dihydrate	CaCl_2_·2H_2_O	29
	Glycine	H_2_NCH_2_CO_2_H	450
	Potassium hydrogen phthalate	(1-(HO_2_C)-2-(CO_2_K)-C_6_H_4_)	4085
	Alkylbenzyldimethylammonium chloride	-	50
Low-calcium Gamble’s solution	Sodium chloride	NaCl	6600
	Sodium bicarbonate	NaHCO_3_	2703
	Calcium chloride	CaCl_2_	22
	Sodium phosphate dibasic dodecahydrate	Na_2_HPO_4_·12H_2_O	358
	Sodium sulphate anhydrous	Na_2_SO_4_	79
	Magnesium chloride hexahydrate	MgCl·6H_2_O	212
	Glycine	H_2_NCH_2_CO_2_H	118
	Sodium citrate dihydrate	Na_3_C_6_H_5_O_7_ ·2H_2_O	153
	Sodium tartrate dihydrate	Na_2_C_4_H_4_O_6_ ·2H_2_O	180
	Sodium pyruvate	C_3_H_3_NaO_3_	172
	Sodium lactate	C_3_H_5_NaO_3_	175

**Table 3:** The following ICP-MS parameters were used to analyze the dissolved fractions of the TiO_2_ (NM-104) and ZnO (NM-110 and NM-111) in phagolysosomal fluid simulant (PSF) and low-calcium Gamble’s solution. The table presents the monitored isotopes related to the nanomaterial, the choice of internal standard, and the limit of detection.

Parameters (Unit)	TiO_2_ (NM-104) Dissolution in PSF	TiO_2_ (NM-104) Dissolution in Gamble’s	ZnO (NM-110 and NM-111) Dissolution in PSF	ZnO (NM-110 and NM-111) Dissolution Gamble’s
Nebulizer gas flow rate (L/min)	0.89	1.06	1.02	1.04
Auxiliary gas flow rate (L/min)	0.80	0.80	0.80	0.80
Helium cell gas flow rate (L/min)	4.35	4.58	No cell gas	No cell gas
Monitored isotopes (*m*/*z*)	^27^Al and ^48^Ti	^27^Al and ^48^Ti	^64^Zn	^64^Zn
Internal standard (*m*/*z*)	^103^Rh	^103^Rh	^103^Rh	^103^Rh
Limit of detection	Al: 19 µg/L, Ti: 3.2 µg/L	Al: 22 µg/L, Ti: 1.2 µg/L	10 µg/L	5.4 µg/L
Dilution factor for ICP-MS analysis	×10	×4	×1000	×200

**Table 4:** Bovine serum albumin adsorption to TiO_2_ (NM-104) (n = 4) and ZnO (NM-110 and NM-111) (n = 2).

Particle Concentration (mg/mL)	Sample	µg BSA/mg NM	Percent Adsorbed of the BSA Added	Literature Values µg BSA/mg NM	Literature Values Percent Adsorbed of the BSA Added
2.56	TiO_2_ (NM-104)	131.8 ± 22.0	69	143.7 ± 12.9 ^#^	66 ^#^
2.56	ZnO (NM-110)	138.3 ± 18.4	67	151.8 ± 27.0 ^ⱡ^	69 ^ⱡ^
2.56	ZnO (NM-111)	88.3 ± 27.1	43	292.8 ± 41.5 ^ⱡ^	40 ^ⱡ^

^#^ Guiot and Spalla (2013) [19]. ^ⱡ^ From Da Silva et al. (2019a) [17].

**Table 5:** Z-average (Z_ave_), zeta potential (Z_pot_), and polydispersity index (PDI) of ZnO (NM-110 and NM-111) and TiO_2_ (NM-104) in pre-dispersions made in water, phagolysosomal fluid simulant (PSF), and low-calcium Gamble’s solution. The values were compared to the literature average agglomeration size (D_aggr_) listed in Table 1 and benchmark Z-averages (Z_ave, benchmark_). See supplementary (Figures S1–S3) for hydrodynamic size number distribution spectra (TiO_2_ (NM-104, S1, A-F), ZnO (NM-110, S2, A-F), and ZnO (NM-111, S3, A-F). The relative color range shown was made using the lowest (green) and highest (red) size ratio when compared with the D_aggr_ and Z_ave,benchmark_ data.

Test Medium	Nanomaterial	Dispersion Medium	Z_ave_ [nm]	PDI	Z_pot_ [mV]	Z_ave,benchmark_	Z_ave_/D_aggr_	Z_ave_/Z_ave,benchmark_
Low-calcium Gamble’s solution	TiO_2_ (NM-104)	Water	157.8 ± 2.3	0.24 ± 0.01	14.9 ± 0.7	234 ± 4 ^a^	2.7	0.7
		GS	2827 ± 1895	0.88 ± 0.3	14.9 + 0.7		48.3	12.1
		0.05% BSA	724.0 ± 160.2	0.74 ± 0.1	−0.8 ± 1.0		12.4	3.1
	ZnO (NM-110)	Water	225.3 ± 2.2	0.15 ± 0.02	30.7 ± 0.6	233.1 ± 7.3 ^b^	2.0	1.0
		GS	1824 ± 343	1.0 ± 0.0	−16.2 ± 0.6		16.0	7.8
		0.05% BSA	250.6 ± 1.1	0.14 ± 0.02	−13.4 ± 0.3		2.2	1.1
	ZnO (NM-111)	Water	735.1 ± 97.0	0.37 ± 0.07	12.5 ± 0.5	247.4 ± 4.9 ^b^	6.9	3.0
		GS	3578 ± 303	0.31 ± 0.1	−13.1 ± 1.0		33.8	14.5
		0.05% BSA	278.9 ± 2.9	0.16 ± 0.02	−14.5 ± 0.5		2.6	1.1
PSF	TiO_2_ (NM-104)	Water	162.9 ± 1.9	0.24 ± 0.01	13.4 ± 0.5	234 ± 4 ^a^	2.8	0.7
		PSF	3448 ± 2283	0.93 ± 0.2	0.6 ± 0.8		58.9	14.7
		0.05% BSA	366.7 ± 153.7	0.30 ± 0.09	−1.3 ± 0.4		6.3	1.6
	ZnO (NM-110)	Water	257.0 ± 1.6	0.16 ± 0.02	24.6 ± 0.6	233.1 ± 7.3 ^b^	2.3	1.1
		PSF	3989 ± 820	1.0 ± 0.0	−15.0 ± 2.6		35.0	17.1
		0.05% BSA	247.1 ± 2.5	0.30 ± 0.09	−14.6 ± 0.6		2.2	1.1
	ZnO (NM-111)	Water	868.1 ± 118.5	0.35 ± 0.07	12.0 ± 0.3	247.4 ± 4.9 ^b^	8.2	3.5
		PSF	5410 ± 680	0.61 ± 0.06	−13.4 ± 2.3		51.0	21.9
		0.05% BSA	275.9 ± 2.6	0.15 ± 0.02	-16.7 ± 0.8		2.6	1.1

^a^ From Jensen et al. [10]. ^b^ From Meija et al. [32].

**Table 6:** *p*-values for interaction between type of dispersion medium and time (t_0_, t_1_, t_2_, t_4_, and t_24_) for dissolution testing conducted in low-calcium Gamble’s solution and PSF. A = 0.05 was used as level of significance (highlighted in bold).

Interaction	TiO_2_ (NM-104)	ZnO (NM-110)	ZnO (NM-111)
Gamble’s solution vs. water	0.5233	0.3792	0.2858
Gamble’s solution vs. 0.05% BSA	**0.0168**	0.3534	**<0.0001**
Water vs. 0.05% BSA	**0.0040**	0.3909	0.0711
PSF vs. Water	0.6167	0.5987	**0.0081**
PSF vs. 0.05% BSA	**<0.0001**	**0.0267**	**0.0023**
Water vs. 0.05% BSA	**<0.0001**	**0.0141**	**0.0011**

**Table 7:** Overview of the initial concentrations (S@t_i_) measured at t_0_ + 25 min (t=0.4 h), the initial dissolutions rate, ${\left(\frac{dC_{A}}{dt}\right)}_{t=0}$ and surface area dissolution rates, ${\left(\frac{dC\left(SSA\right)}{dt}\right)}_{t=0}$, determined from the batch reactor setup ND: not determined, quick dissolution. The results are presented as average values ± standard deviation (n = 3 batch reactors).

		Test Medium: Gamble’s Solution			Test Medium: PSF		
		Dispersion Medium			Dispersion Medium		
Nanomaterial	Initial Concentration and Dissolution Rates	Gamble’s Solution	Water	0.05% BSA	PSF	Water	0.05% BSA
TiO_2_ (NM-104), Al_2_O_3_ coating	S@t_i_ (mg/L)	0.097 ± 2.04 × 10^−3^	0.096 ± 7.94 × 10^−3^	0.092 ± 1.65 × 10^−3^	0.139 ± 2.39 × 10^−3^	0.0965 ± 2.91 × 10^−3^	0.069 ± 4.0 × 10^−3^
	${\left(\frac{dC_{A}}{dt}\right)}_{t=0}$, (mg/L/h)	0.171 ± 0.064	0.126 ± 0.018	0.160 ± 0.038	0.230 ± 0.031	0.220 ± 0.014	0.096 ± 0.002
	${\left(\frac{dC\left(SSA\right)}{dt}\right)}_{t=0}$, (cm^2^/L/sec)	0.027 ± 0.010	0.020 ± 2.8 × 10^−3^	0.025 ± 5.92 × 10^−3^	0.036 ± 4.8 × 10^−3^	0.034 ± 2.26 × 10^−3^	0.015 ± 2.73 × 10^−4^
ZnO (NM-110)	S@t_i_ (mg/L)	22.3 ± 2.5	16.4 ± 0.4	10.7 ± 0.5	116.3 ± 7.3	125.0 ± 0.9	108.1 ± 2.3
	${\left(\frac{dC_{A}}{dt}\right)}_{t=0}$, (mg/L/h)	2.72 ± 2.59	0.720 ± 0.191	2.04 ± 0.22	ND	ND	ND
	${\left(\frac{dC\left(SSA\right)}{dt}\right)}_{t=0}$, (cm^2^/L/sec)	0.094 ± 0.089	0.025± 6.6 × 10^−3^	0.070 ± 7.74 × 10^−3^	ND	ND	ND
ZnO (NM-111)	S@t_i_ (mg/L)	4.83 ± 0.27	5.76 ± 0.23	4.10 ± 0.61	112.1 ± 0.8	104.5 ± 3.8	74.67 ± 4.52
	${\left(\frac{dC_{A}}{dt}\right)}_{t=0}$, (mg/L/h)	0.604 ± 0.582	2.09 ± 1.71	1.95 ± 0.26	ND	ND	ND
	${\left(\frac{dC\left(SSA\right)}{dt}\right)}_{t=0}$(cm^2^/L/sec)	0.025 ± 0.024	0.088± 0.072	0.082 ± 0.011	ND	ND	ND

## Data Availability

The data are available from the eNanoMapper database when the embargo of the EU project PATROLS is lifted in October 2023; http://www.enanomapper.net/data (accessed on 9 October 2021).

## Supplementary Materials

The following are available online at https://www.mdpi.com/article/10.3390/nano12030566/s1, Figure S1: Hydrodynamic size distribution of TiO_2_ (NM-104), Figure S2: Hydrodynamic size distribution of ZnO (NM-110), Figure S3: Hydrodynamic size distribution of ZnO (NM-111).
