# Supplementary material for: Influence of Pre-Dispersion Media on the Batch Reactor Dissolution Behavior of Al2O3 Coated TiO2 (NM-104) and Two ZnO (NM-110 and NM-111) Nanomaterials in Biologically Relevant Test Media
Source: Nanomaterials (Basel). 2022 Feb 7;12(3):566. doi: 10.3390/nano12030566 (PMC8840498; doi:10.3390/nano12030566)
Supplement: Supplementary file 1 [file nanomaterials-12-00566-s001.zip › nanomaterials-1435596-supplementary.pdf]

nanomaterials

Supplementary materials

Influence of Pre-Dispersion Media on the Batch Reactor Dissolution Behavior of Al_2O_3 Coated TiO_2 (NM-104) and Two ZnO (NM-110 and NM-111) Nanomaterials in Biologically Relevant Test Media

Else Holmfred [1,2,*], Jens J. Sloth [2], Katrin Loeschner [2], and Keld Alstrup Jensen [1,*]

[1] National Research Center for the Working Environment, 2100 Copenhagen, Denmark
[2] Research Group for Analytical Food Chemistry, Division of Food Technology, National Food Institute, Technical University of Denmark, 2800 Kongens Lyngby, Denmark; jjsl@food.dtu.dk (J.J.S.); kals@food.dtu.dk (K.L.)
* Correspondence: elshol@food.dtu.dk (E.H.); kaj@nfa.dk (K.A.J.)

Citation: Holmfred, E.; Sloth, J.J.; Loeschner, K.; Jensen, K.A. Influence of Pre-Dispersion Media on the Batch Reactor Dissolution Behavior of Al_2O_3 Coated TiO_2 (NM-104) and Two ZnO (NM-110 and NM-111) Nanomaterials in Biologically Relevant Test Media. *Nanomaterials* **2022**, *12*, 566. https://doi.org/10.3390/nano12030566

Academic Editor: Andrea Hartwig

Received: 9 October 2021
Accepted: 30 January 2022
Published: 7 February 2022

Publisher's Note: MDPI stays neutral with regard to jurisdictional claims in published maps and institutional affiliations.

Copyright: © 2022 by the authors. Licensee MDPI, Basel, Switzerland. This article is an open access article distributed under the terms and conditions of the Creative Commons Attribution (CC BY) license (https://creativecommons.org/licenses/by/4.0/).

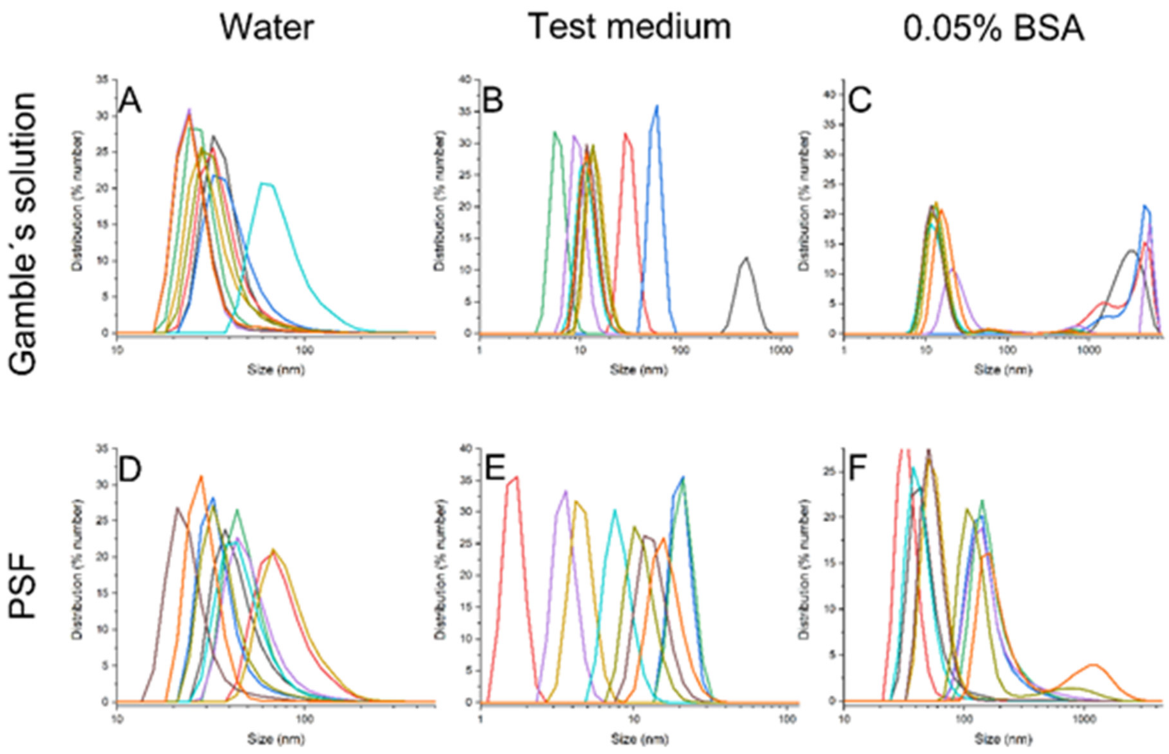

Figure S1. Hydrodynamic size distribution of TiO_2 (NM-104). Dispersed in (**A**) water; (**B**) low-calcium Gamble's solution; (**C**) 0.05% BSA; (**D**) water; (**E**) phagolysosomal simulant fluid (PSF); (**F**) 0.05% BSA. The dissolution behavior A-C was further tested in low-calcium Gamble's solution and D-F in PSF.

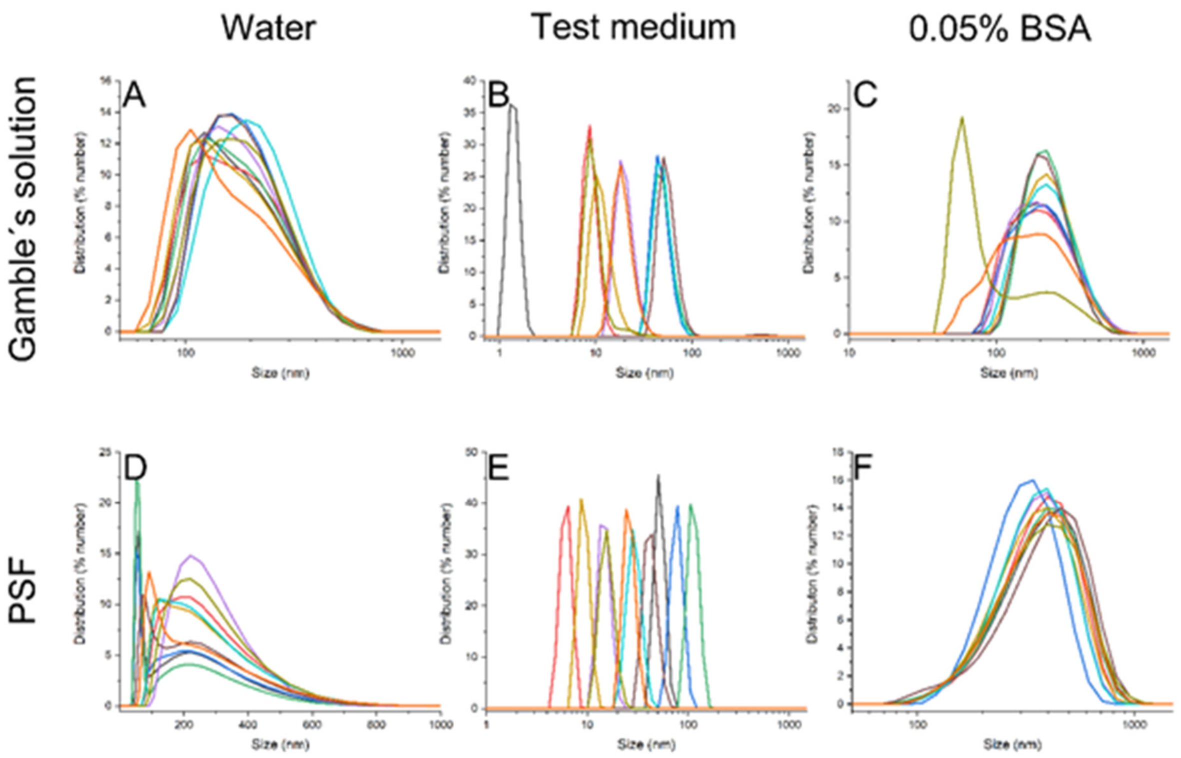

Figure S2. Hydrodynamic size distribution of ZnO (NM-110). Dispersed in (**A**) water; (**B**) low-calcium Gamble's solution; (**C**) 0.05% BSA; (**D**) water; (**E**) phagolysosomal simulant fluid (PSF); (**F**) 0.05% BSA. The dissolution behavior A-C was further tested in low-calcium Gamble's solution and D-F in PSF.

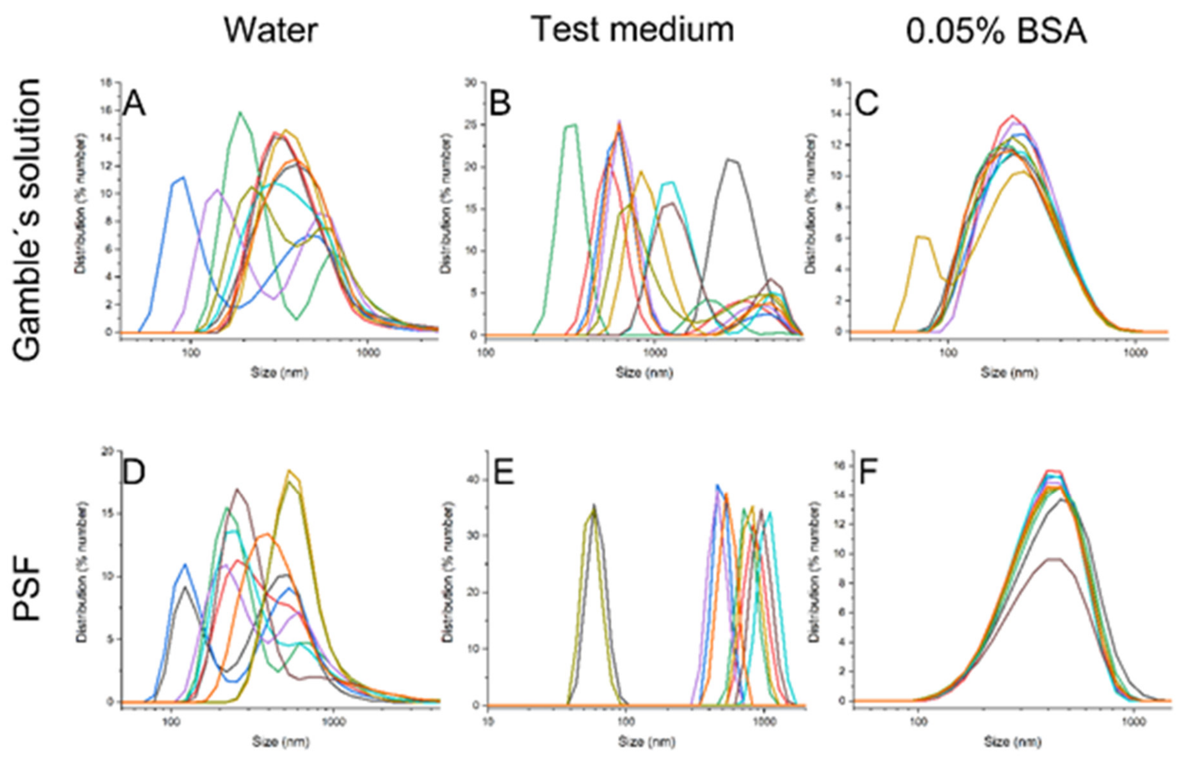

Figure S3. Hydrodynamic size distribution of ZnO (NM-111). Dispersed in (**A**) water; (**B**) low-calcium Gamble's solution; (**C**) 0.05% BSA; (**D**) water; (**E**) phagolysosomal simulant fluid (PSF); (**F**) 0.05% BSA. The dissolution behavior A-C was further tested in low-calcium Gamble's solution and D-F in PSF.
